# Effect of Dietary Strategies on Respiratory Quotient and Its Association with Clinical Parameters and Organ Fat Loss: A Randomized Controlled Trial

**DOI:** 10.3390/nu13072230

**Published:** 2021-06-29

**Authors:** Ariela Goldenshluger, Keren Constantini, Nir Goldstein, Ilan Shelef, Dan Schwarzfuchs, Hila Zelicha, Anat Yaskolka Meir, Gal Tsaban, Yoash Chassidim, Yftach Gepner

**Affiliations:** 1 Department of Epidemiology and Preventive Medicine, School of Public Health, Sackler Faculty of Medicine, and Sylvan Adams Sports Institute, Tel-Aviv University, Tel-Aviv 96678, Israel; arielag@mail.tau.ac.il (A.G.); kconstantini@mail.tau.ac.il (K.C.); goldsnir@tauex.tau.ac.il (N.G.); 2 Radiology Department, Soroka University Medical Center, Beer-Sheva 84101, Israel; shelef@bgu.ac.il; 3 Emergency Medicine Department, Soroka University Medical Center, Beer-Sheva 84101, Israel; dan.nurit@gmail.com; 4 Faculty of Health Sciences, Ben-Gurion University of the Negev, Beer-Sheva 84105, Israel; hila.zelicha@gmail.com (H.Z.); anatyas@gmail.com (A.Y.M.); gtsaban@gmail.com (G.T.); 5 Industrial and Management Department, Sapir College, Sderot 79165, Israel; yoash.chassidim@gmail.com

**Keywords:** respiratory quotient, intra-abdominal fat, weight loss, indirect calorimetry, diet

## Abstract

The relation between changes in respiratory quotient (RQ) following dietary interventions and clinical parameters and body fat pools remains unknown. In this randomized controlled trial, participants with moderate abdominal obesity or/and dyslipidemia (*n* = 159) were randomly assigned to a Mediterranean/low carbohydrate (MED/LC, *n* = 80) or a low fat (LF, *n* = 79) isocaloric weight loss diet and completed a metabolic assessment. Changes in RQ (measured by indirect calorimeter), adipose-tissue pools (MRI), and clinical measurements were assessed at baseline and after 6 months of intervention. An elevated RQ at baseline was significantly associated with increased visceral adipose tissue, hepatic fat, higher levels of insulin and homeostatic insulin resistance. After 6 months, body weight had decreased similarly between the diet groups (−6 ± 6 kg). However, the MED/LC diet, which greatly improved metabolic health, decreased RQ significantly more than the LF diet (−0.022 ± 0.007 vs. −0.002 ± 0.008, *p* = 0.005). Total cholesterol and diastolic blood pressure were independently associated with RQ changes (*p* = 0.045). RQ was positively associated with increased superficial subcutaneous-adipose-tissue but decreased renal sinus, pancreatic, and intramuscular fats after adjusting for confounders. Fasting RQ may reflect differences in metabolic characteristics between subjects affecting their potential individual response to the diet.

## 1. Introduction

Obesity is related to metabolic [1,2] and cardiovascular risks [3,4] through excessive weight and adiposity [1]. Specifically, visceral adipose tissue (VAT) has been associated with obesity-related health risks [1]. Previous reports have suggested that dietary intervention, either alone [5] or combined with exercise [2,6], can benefit health, irrespective of weight loss per se, by reducing VAT [2,5,7,8], hepatic fat [5,7], and intrapericardial fat [5], and by mobilizing additional internal stored fat [5,7,8,9].

A Mediterranean diet was found to be more strongly associated with health benefits, including cardiovascular disease risk reduction [10,11,12,13,14], and prevention of type 2 diabetes [15], than other regimes, such as a low fat diet [11], the dietary approach to stop hypertension (DASH) diet, or the Paleolithic, or Nordic diets [12,13]. In our previous, DIRECT dietary randomized control trial intervention, we found that adherence to Mediterranean or low-carbohydrate diets improved cardiometabolic markers and reversed changes in the volume of the carotid wall [16]. We also reported a reduction in ectopic fat depots, [5] and intrahepatic fat, which was independently associated with improved blood metabolic parameters [5,7,17]. Some evidence suggests that the health benefits of the Mediterranean diet are mediated by a limitation in carbohydrate intake [18], especially when it is replaced by unsaturated fats and specifically by polyunsaturated fats [18,19].

Respiratory quotient (RQ) is the ratio between exhaled CO_2_ and inhaled O_2_ [20], and reflects the contribution of different macronutrients as energy sources [20]. At rest, RQ ranges between 0.7 and 1, where a value of 1 implicates carbohydrates as the main energy source, and 0.7 reflects the predominant use of fat [20]. Variations in macronutrient intake (fats versus carbohydrates) during the day have been related to changes in oxidation rates, suggesting a relationship between RQ and the concentration of substrates (e.g., glucose and fatty acids) in the plasma [21]. Additionally, higher postprandial RQ was positively correlated with a higher glycemic index of meals [22]. However, there is little evidence linking changes in RQ to diet type as a whole, as opposed to individual macronutrients [23,24,25]. Tea has been found to affect RQ differently along daily hours and to reduce RQ after sleeping and fasting but not immediately after tea consumption [26]. In a fasting state, a higher RQ has been associated with higher weight gain and fat storage [22,27], contrarily to an increase in RQ following weight loss, in subjects with previous obesity [28]. The associations between RQ and weight change are inconsistent [27,28,29,30,31]. To the best of our knowledge, the question of whether changes in fasted state RQ following dietary interventions influence clinical parameters remains unanswered. The present study was designed to assess the impact of changes in RQ on clinical parameters and body fat pools, during weight loss and to evaluate the contribution of different diets to RQ change.

## 2. Materials and Methods

### 2.1. Study Population

**Figure 1:**
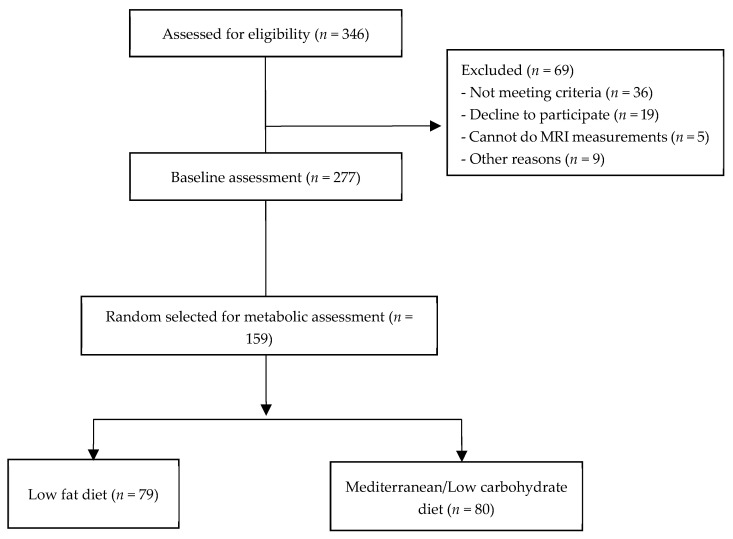
Flow chart of the CENTRAL metabolic sub-study.

This is a sub-study extracted from a large one-phase 18-month randomized controlled lifestyle interventions trial (CENTRAL trial; ClinicalTrials.gov Identifier: NCT01530724) [5]. All 277 study participants recruited for baseline assessment had moderate abdominal obesity or/and dyslipidemia and worked in the same isolated workplace with a monitored provided lunch. A random sub-group of 159 individuals were randomly assigned to consume either a Mediterranean/low carbohydrate (MED/LC, *n* = 80) or a low fat (LF, *n* = 79) iso-caloric weight loss intervention diet for 6 months (Figure 1) and completed a metabolic assessment. Subjects were eligible for this study if they had abdominal obesity [waist circumference (WC) >40 inches (102 cm) for men and >35 inches (88 cm) for women], or triglycerides (TG) ≥150 mg/dL, and high density lipoprotein cholesterol (HDL-c) <40 mg/dL for men and <50 mg/dL for women. Exclusion criteria included pregnancy or lactation; serum creatinine ≥2 mg/dL; impaired liver function (≥3 times the upper normal level of ALT and AST enzymes); active cancer; high level of physical activity (>3 h/week); inability to take part in physical activity; and/or active participation in another trial. The study protocol was approved by the Medical Ethics Board and the Helsinki Committee of the Soroka University Medical Center and did not change after trial commencement. Participants provided written informed consent and received no financial compensation.

### 2.2. Dietary Intervention

Both diets were designed to achieve a moderate, long-term, weight loss with restricted intake of *trans*-fats and refined carbohydrates, and an increased intake of vegetables [5]. Lunch was provided exclusively by the workplace cafeteria during the work week, and a registered dietitian worked closely with the kitchen staff to adjust the menu for the specific diet groups. Participants also met with a registered dietitian on a weekly basis during the first month of intervention, and monthly thereafter. During these 90-min nutritional sessions, participants received personalized instructions regarding their diet. Both diets aimed for an energy intake of 1500 kcal/day for women and 1800 kcal/day for men. The MED/LC diet combined the Mediterranean and low-carbohydrate diets [16] and restricted carbohydrate intake to less than 40 g/day in the first two months, with a gradual increase thereafter up to 70 g/day. In addition, participants in the MED/LC diet group were provided 28 g of walnuts/day (160 Kcal/84% fat, mostly PUFA (omega-3 α-linolenic acid)) starting from the third month. In general, the MED/LC diet emphasized a relatively high protein and fat intake, mostly from vegetarian sources, according to the Mediterranean diet. It was rich in vegetables and legumes, and low in red meat, with poultry and fish replacing beef and lamb. The LF diet, based on the American Heart Association guidelines [32] was limited to a fat intake of 30% of total calories, with up to 10% of saturated fat, and up to 300 mg/day of cholesterol and was designed to increase dietary fiber. Additionally, the diet encouraged eating whole grains, vegetables, fruits, and legumes while limiting the consumption of additional fats, sweets, and high-fat snacks. Adherence to the diets was assessed by monitoring attendance of the weekly (during the first month) and monthly (thereafter) nutritional sessions, and was quantified via a self-administered validated electronic, 127-item food-frequency questionnaire at baseline and after 6 months. Text messages with reminders were sent at intervals to the participants in order to update them and motivate adherence to the diets on specific occasions (such as before and after holidays).

### 2.3. Indirect Calorimetry

RQ was measured by indirect calorimetry (QUARK RMR by COSMED, Rome, Italy) at baseline and after 6 months of intervention, using a ventilated canopy. Two hundred and seventy-seven participants had a valid RQ measurement at baseline. Gas calibration was performed before each test and turbine calibration was implemented every day, according to the manufacturer’s instructions. After a 10 min rest, subjects lay awake in a supine position during the 20 min of measurement. All measurements were performed in the same quiet room at a stable temperature (22–24 °C), and participants watched relaxing channels on TV in order to avoid falling asleep or making extreme movements. All measurements were performed at the same time along the day for each participant. RQ was calculated from the gas exchange (exhaled CO_2_ and inhaled O_2_) by applying the Weir equation [33]. The first 4 min (steady states phase) were excluded from the analysis and the mean of the subsequent 16 min was defined as the RQ. Prior to this measurement, participants were asked to fast for at least 4 h and to avoid alcohol, caffeine, smoking, and physical activity for 8 h prior to the test [34]. Changes in RQ were calculated as the difference between post (6 months after intervention) and baseline measurements. 

### 2.4. Magnetic Resonance Imaging (MRI)

MRI was performed using a 3-Tesla magnet (Ingenia 3.0 T, Philips Healthcare, Best, The Netherlands) at baseline and after 6 months. The scanner utilized a 3-dimensional modified DIXON imaging technique without gaps (2 mm thickness and 2 mm of spacing) [5] and a fast-low-angle shot sequence with a multi-echo 2-excitation pulse sequence for phase-sensitive encoding of fat and water signals (repetition time, 3.6 ms; echo time 1, 1.19 ms; echo time 2, 2.3 ms; FOV 520 × 440 × 80 mm; 2 × 1.4 × 1 mm voxel size) [5]. Four images of the phantoms were generated, including in-phase, out-phase, fat phase, and water phase. In all simultaneous fat depots quantification and comparisons, observers were blinded to time point and group treatment. All fat depots were assessed by one or two raters. The Inter-observer and intra-observer correlations were >0.96 (*p* < 0.001) for all measured fat storage pools, and 0.95, *p* < 0.001 for pericardial fat volume. Adipose tissue sub-depot analyses were performed while blinded to time-point and treatment group. The reliability of measurements between technicians was evaluated by comparing 28 images (r = 0.998).

Abdominal fat depots: We quantified abdominal fat using the MATLAB-based semi-automatic software [5,35]. We drew a continuous line over the fascia superficialis to differentiate between the deep-SAT and superficial-SAT, and calculated mean VAT, deep-SAT and superficial-SAT from three axial slices: L5-S1, L4-L5 and L2-L3. Quantification of the fat mass regions included the area (cm^2^) of each sub-fat type. Hepatic fat content: we quantified the percentage of hepatic fat using PRIDE software from Philips Medical Systems. We calculated mean percentage from four 2D slices (3 cm intervals divided into quarters) by utilizing the region of interest (ROI) approach, which is based on measurements of tissue densities (fat/fat + water) using the fat ratio calculation [7]. We divided each slice into quarters and chose ROIs in each of the four quarters in order to represent the entire liver. We determined the mean percentage of fat for each slice and quarter and then calculated the mean percentage of fat in the liver as a whole. Pancreatic fat: pancreatic fat percentage was calculated from fat-phase images (fat/fat + water), by taking the average of three successive 2D slices, each including all pancreatic regions. The MRI method was validated against 1H-MRS and readily distinguished pancreatic parenchymal tissue from VAT [36]. Renal sinus fat: renal-sinus fat was analyzed using the semi-automatic MATLAB-based software. We acquired the amount of renal-sinus fat area (cm^2^) from the middle axial slice of each kidney at the level of the 1st–2nd lumbar vertebra, by calculating the area of the kidney by polygon. As the right kidney sits slightly lower than the left to accommodate the liver, we defined the slices accordingly [37]. Femoral intermuscular adipose tissue: We quantified femoral intermuscular adipose tissue (femoral-IMAT) by calculating the area (cm^2^) of a single axial slice from mid-thigh of the right leg, between the femoral head to medial and lateral condyle. IMAT area was based on measurements of tissue densities (fat/fat + water) using the fat ratio calculation by the mDIXON sequence [38]. Intramuscular triglycerides (IMTG) content was quantified by utilizing the region of interest (ROI) technique. This method is based on a comparison of tissue density (fat/fat + water) in the selected regions. Using semi-automatic PRIDE software from Philips Medical Systems, we analyzed the middle hip 2D image in the central area of four muscles: rectus femoris, vastus lateralis, adductor magnus and semitendinosus. The mean percentage of IMTG was calculated by using all the values of each ROI [39].

### 2.5. Clinical and Anthropometric Outcomes

Height was measured using a standard wall-mounted stadiometer. Waist circumference was measured half-way between the last rib and the iliac crest. Fasting blood samples were centrifuged and stored at −80 °C. All biomarkers were assayed in the Leipzig University laboratories, Germany. Blood samples were analyzed as described previously [5]. Homeostasis model assessments of insulin resistance (HOMA-IR) were calculated using the HOMA Calculator v2.2.3. 

### 2.6. Statistical Analysis

The original sample size was estimated based on our primary aim, which was changes in VAT [5], and was based on a previous study, where we found a significant 12.8% change in VAT (*p* < 0.05). A linear regression model adjusted for age, sex, and baseline body mass index (BMI) was used to test the association between baseline RQ, and baseline body fat, anthropometric parameters, cardiometabolic markers, and cytokines. In order to examine whether any effects of diet intervention on the cardio-metabolic profile depend on baseline RQ values (i.e., high vs. low RQ), we performed a two-way ANOVA analysis that included diet (MED/LC and LF) and baseline RQ as independent variables. Baseline RQ was categorized as “low RQ” for those individuals with values below the median RQ, and “high RQ” for those with RQ above the median. For factors showing a significant interaction effect, we performed an analysis based on stratification of baseline RQ (high RQ and Low RQ). 

A multivariate linear regression model was performed to assess the associations between 6-months changes in RQ (RQ increase/decrease) and the above-mentioned variables, adjusted for potential confounders (age, gender, diet group, baseline RQ, and 6-month change in BMI). All statistical analyses were two sided with a significance of *p* < 0.05. Statistical analysis was carried out using the SPSS version 25 (SPSS Inc., Chicago, IL, USA) software.

## 3. Results

**Figure 2:**
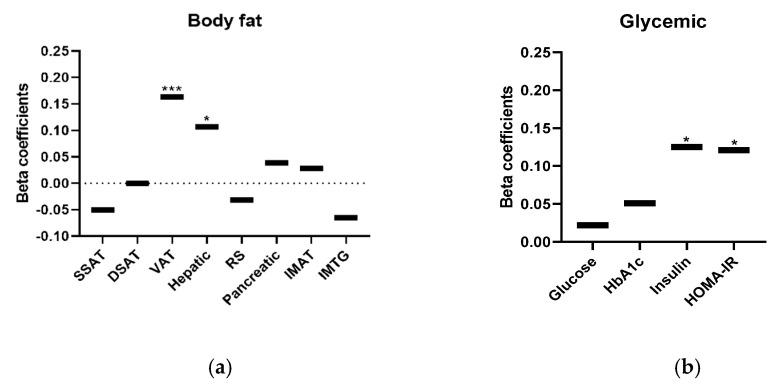

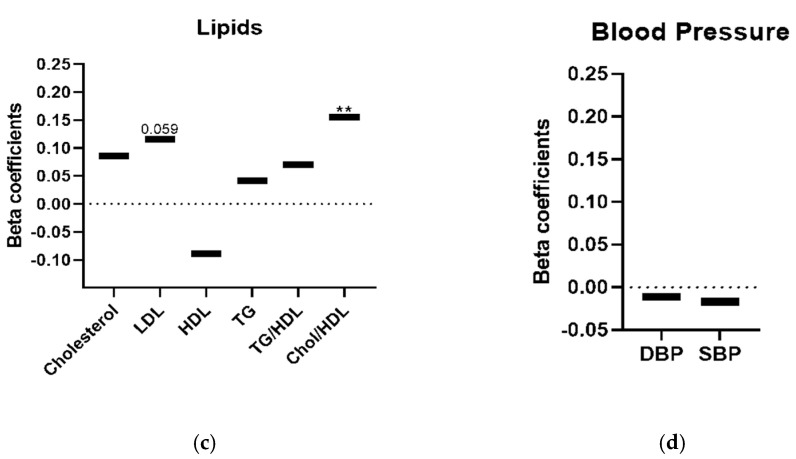
(**a**–**d**). Baseline associations between cardio-metabolic parameters and body fat and RQ. Linear regression model adjusted for age, sex, and baseline BMI. Amount and direction of association between baseline RQ values and biomarkers are represented by β-standardized coefficient. *n* = 277. * *p* < 0.05; ** *p* < 0.01; *** *p* < 0.001. Abbreviations: DBP—diastolic blood pressure; DSAT—deep subcutaneous adipose tissue; HbA1C—glycated hemoglobin; HOMA-IR—homeostatic model of insulin resistance; HDL—high-density lipoprotein cholesterol; IMAT—intermuscular adipose tissue; IMTG intramuscular triglycerides; LDL—low-density lipoprotein; RQ—respiratory quotient; RS—renal sinus; SBP—systolic blood pressure; SSAT—superficial subcutaneous adipose tissue; TG—triglycerides; VAT—visceral adipose tissue.

Baseline associations for the 277 participants (age 48 ± 9 years, body weight 91 ± 13 kg) revealed that RQ was positively associated with VAT area (β = 0.163, *p* < 0.0001), hepatic fat content (β = 0.107, *p* = 0.049, Figure 2a) and higher insulin (β = 0.125, *p* = 0.029 Figure 2b). Elevated RQ was associated with higher lipid (Figure 2c) and adipokine ratios after controlling for age, sex, and baseline BMI.

**Figure 3:**
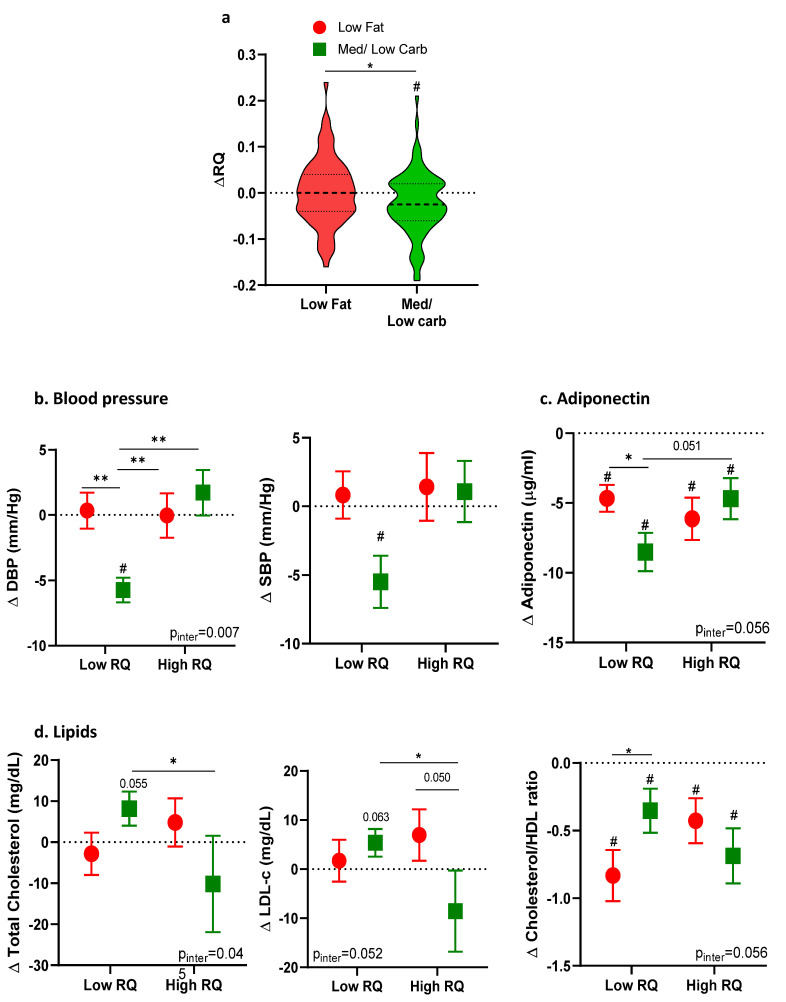
(**a**) RQ changes after 6 months intervention across diet groups. (**b**–**d**) Changes in cardiometabolic parameters and adiponectin across RQ levels stratification and diet groups. (*n* = 159). Stratification was calculated following a significant or a trend in a two-way ANOVA analysis that included diet and RQ as independent variables. Significant differences between groups are represented by * *p* < 0.05; ** *p* < 0.01; # significant difference between baseline to 6 months *p* < 0.05.Abbreviations: DSAT—deep subcutaneous adipose tissue; DBP—diastolic blood pressure; HbA1C—Glycated hemoglobin; RQ—respiratory quotient; SBP—systolic blood pressure; SSAT—superficial subcutaneous adipose tissue; VAT—Visceral adipose tissue.

Baseline characteristics for participants who completed the intervention diets and full metabolic assessment (*n* = 159) are summarized in Table 1. Participants’ average age was 47.7 years and BMI was 31.1 ± 4.1 kg/m^2^. At baseline, RQ was similar between diet groups (LF: 0.80 ± 0.04; MED/LC 0.81 ± 0.05, 95%CI −0.023 to 0.006 *p* = 0.299) and both groups consumed similar amounts of kilocalories, carbohydrates, and protein, with a minor difference (1.6%) in fat intake. The characteristics of the members of the random sample who underwent metabolic assessment after 6 months (*n* = 159) did not differ from participants who were measured only at baseline (Appendix A Table A1). After 6 months of intervention, RQ decreased only in the MED/LC diet group. The decrease was 7.3% of the total range of change, and was significantly different from the change in RQ in those who consumed the LF diet (mean change −0.022 ± 0.007 and 0.002 ± 0.008 for the MED/LC and LF diet groups respectively, mean difference −0.024, 95%CI 0.002 to 0.04, *p* = 0.005, Figure 3a). 

After 6 months, both diet groups had reduced their caloric consumption (mean change −775 ± 1599 Kcal and −933 ± 1072 Kcal respectively, mean difference −158 Kcal, *p* = 0.47), but the macronutrient constituents differed between the MED/LC and LF diets: %carbohydrates (31.8 ± 9.7% vs. 43.7 ± 8.2%, mean difference 11.9%, *p* < 0.001 respectively), %fat (44.0 ± 6.8% vs. 34.7 ± 5.3%, mean difference −9.2%, *p* < 0.001 respectively), and %protein (25.1 ± 4.1% vs. 22.5 ± 3.5%, mean difference −2.9%, *p* < 0.001 respectively). There was a significant interaction between RQ and the diet group for total cholesterol and diastolic blood pressure (*p* = 0.045 and *p* = 0.007 for the MED/LC and LF diets respectively), although a statistically significant reduction in both diastolic and systolic blood pressure (Figure 3b) was only observed in participants in the low RQ group on the MED/LC diet (*p* < 0.001, *p* = 0.001, respectively). Although the levels of HDL-c and the glycemic profile improved similarly between diets, there was a trend for interaction between diet type and baseline RQ for total cholesterol/HDL-c (*p* = 0.056) and low density lipoprotein cholesterol (LDL-c, *p* = 0.052). LDL-c was lower in participants with a high RQ on the MED/LC diet (*p* = 0.045), and the ratio of Chol/HDL-c was lower in the Low RQ group on the LF diet than on the MED/LC diet (*p* = 0.032, Figure 3d). While adiponectin was reduced in all groups, the low RQ group on the MED/LC diet exhibited a greater change in adiponectin than the LF and a trend for the high RQ groups (*p* = 0.022 and *p* = 0.051 respectively, Figure 3c, *p* for interaction = 0.056). Leptin was reduced similarly in all diet groups (mean difference −9.1 ng/mL for the MED/LC diet, and −7.6 ng/mL for the LF diet, *p* > 0.05).

**Figure 4:**
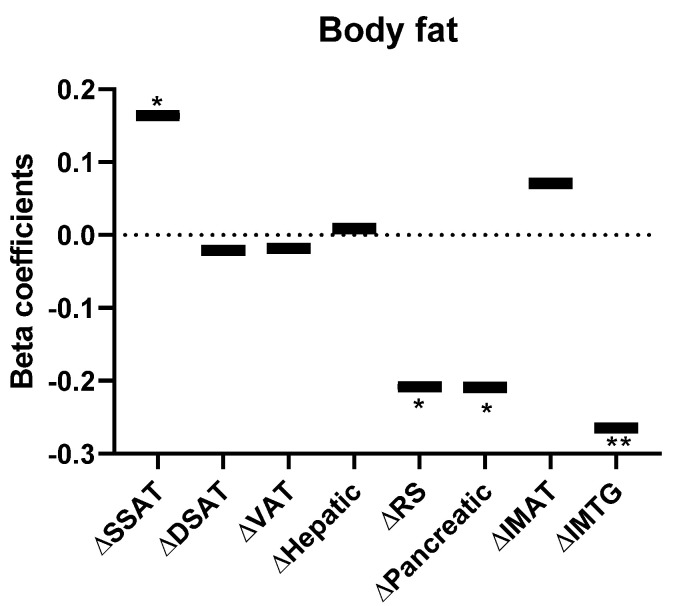
Association between body fat and 6 month changes in RQ (decreased/increased, *n* = 159). Multivariate linear regression model was used to assess the association between body fat and RQ changes (increased/decreased), adjusted for age, sex, diet group, baseline RQ, and changes in BMI. Standardized-Beta coefficients are presented for each variable. * *p* < 0.05; ** *p* < 0.01. Abbreviations: DSAT—deep subcutaneous adipose tissue; IMAT—intermuscular adipose tissue; IMTG intramuscular triglycerides; RQ—respiratory quotient; RS—renal sinus; SSAT—superficial subcutaneous adipose tissue; VAT—Visceral adipose tissue.

**Figure A1:**
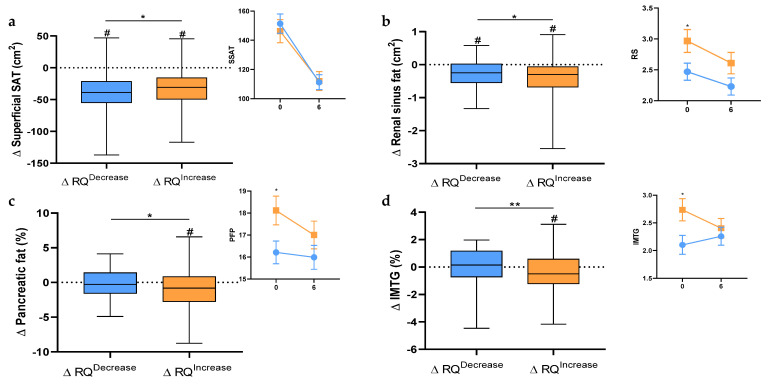
(**a**–**d**) Changes in body fat among people with a decrease (blue boxes) and increase (orange boxes) in RQ. Inserts: Body fat at baseline and after 6 months among people with decreased (blue) or increased RQ (orange). Un-paired samples *t*-test was used to compare between groups * *p* < 0.05; ** *p* < 0.01; paired samples *t*-test was used for comparisons between time-points # significant difference between baseline to 6 months. Abbreviations: IMTG intramuscular triglycerides; RQ-respiratory quotient; SAT—subcutaneous adipose tissue.

Finally, we asked whether increases or decreases in RQ (RQ^increase^ and RQ^decrease^, respectively) could be associated with changes in clinical parameters. A multivariate model adjusted for age, sex, baseline RQ, diet and changes in BMI, revealed that various measures of body fat were affected by the change in RQ (Figure 4). All participants lost superficial SAT area, but those with RQ^decrease^ lost significantly more (−41.05 ± 3.27 cm^2^) than participants with RQ^increase^ (−34.11 ± 3.75 cm^2^, *p* = 0.047, Appendix A Figure A1). Participants in the RQ^increase^ group lost more RS and pancreatic fat than those with RQ^decrease^ (−0.15 ± 0.02 vs. −0.12 ± 0.02 and −1.11 ± 0.34 vs. −0.24 ± 0.24 kg, respectively). Intramuscular triglycerides slightly increased in participants in the RQ^decrease^ group (0.14 ± 0.13 kg), and decreased in the RQ^increase^ group (−0.33 ± 0.16 kg, Appendix A Figure A1).

## 4. Discussion

The results of the present study revealed that elevated RQ is associated with an adverse cardiometabolic profile, insulin resistance, and ectopic fat. Greater reductions in RQ were found during weight loss intervention by the MED/LC diet than a LF diet. Interestingly, the two types of diets affected clinical parameters differently, according to the participants’ baseline RQ. The parameters affected were related to a reduction in blood pressure, and a decrease in total cholesterol. A trend of interaction was seen for LDL-c and the cholesterol/HDL-c ratio with a differential decrease in adiponectin. In addition, our results revealed that changes in fasted state RQ might reflect different metabolic characteristics, such as insulin sensitivity/resistance and associate with changes in specific fat depots such as superficial SAT, renal sinus, pancreatic fat, and intramuscular triglycerides.

Our observations that a MED/LC diet reduced RQ levels more than a LF diet are in accordance with results obtained in a randomized, crossover feeding study (*n* = 19) following 6 days of restricted carbohydrates vs. dietary fat restriction [40]. In the current study, RQ decreased only in the group following the MED/LC diet where the reduction was 7.3% of total range after 6 months of well-controlled intervention. There was no change (0.66%) in the LF diet group. Such a metabolic shift in carbohydrate utilization may be related to an individual response in insulin regulation and the decrease in body fat pools. In another crossover study, 4 weeks of ketogenic diet (~78% fat) resulted in a decrease of RQ from baseline [23]. However, the effects of different macronutrient compositions in the diet on RQ in participants of healthy weight [41,42,43,44] or those with obesity [43], are somewhat controversial. It has been suggested that in extreme circumstances, substrate oxidation can compensate for a missing substrate if another is dominant. However, it should be noted that such diet experiments are viable in research settings and may not truly reflect real life [41,45]. 

Basal RQ values have been associated with an adverse cardiometabolic profile including elevated VAT, hepatic fat, insulin, and HOMA-IR, as well as Chol/HDL-c and leptin/adiponectin ratios. The literature evaluating RQ and health related outcomes is limited, and mainly consists of reports of observational studies. Previous studies have associated RQ and metabolic outcomes such as hypertriglyceridemia [46,47], and similarly to our results, to insulin function [21,48,49], and hepatosteatosis [50], where higher RQ may increase hepatic fat accumulation and promote liver pathogenesis due to lower fat oxidation [50]. Substrate oxidation has also been associated with vascular outcomes such as systolic blood pressure [51] and subclinical atherosclerosis [52]. In accordance with our results, no significant associations between RQ, BMI, VAT, and fat mass have been observed [46,50]. Unlike our results, a previous large cross sectional study (*n* = 2819) failed to identify any associations between RQ and adiposity indexes [46]. However, significant methodological differences may have contributed to these discrepancies with our results. Longitudinal studies generally associate higher RQ with a greater risk for weight [53,54] and fat mass gain [53] but no association for weight regain after weight loss in subjects with obesity [28].

In this study, we assessed changes in substrate oxidation in response to two different diets: the MED/LC diet and a LF diet. The negative energy balance and weight reduction were similar across the board and may have affected the leptin decrease, which aligns with fat mass loss as described elsewhere [55]. Since RQ measurement was performed in a fasted state, changes in RQ may reflect metabolic differences between individuals, which may affect the response to the different diets. Interestingly, after evaluating the interactions between baseline RQ and diet type, we identified differences in the response of certain cardiometabolic parameters between participants following a similar diet, depending on whether they started the study with a low or high RQ. For example, diastolic blood pressure was significantly reduced only in the lower RQ group on the MED/LC diet. Similarly, a cross sectional study [51] reported a correlation between RQ and blood pressure and suggested that mechanisms for lipid oxidation may activate the renin angiotensin system and thus influence blood pressure [51,56]. This mechanism was supported by a mouse study that demonstrated an increase in fat oxidation and a reduction in fat mass when the angiotensin-II receptor was absent [57]. Our study also detected differential results for total cholesterol and trends for LDL, the cholesterol/HDL-c ratio, and adiponectin. 

We suggest that the interpersonal variance in RQ may reflect the metabolic state, in which higher RQ associates with reduced insulin sensitivity. In addition, differences in RQ may affect the individual response to diet. Accordingly, the response to the Mediterranean diet may vary interpersonally, according to the baseline RQ.

Further interventional studies are needed to understand the physiological origins of interpersonal variations in response to diet type. 

Limitations: Although this sub-study of the CENTRAL study involved a longitudinal metabolic analysis of only part of the main sample, most characteristics were comparable to those of the original study population (see Appendix A). Representation of women in our study was low (14.5%), reflecting the workplace distribution, and may limit the generalizability of the result for women. For this reason, the multivariate models were adjusted for sex to reduce any confounding effect. In addition, a review reported no gender differences in substrate oxidation among studies [41]. Further research is needed to investigate this key metabolic phenomenon in more detail. 

Although this study lacks a food quotient calculation, we assume that after a long-term intervention, participants have adapted to their diet and that there may have been a variety of changes made within the same group. After six months of diet intervention, the main weight loss has already occurred. Therefore, changes in fasting RQ may reflect principally metabolic characteristics of the participants rather than response to diet per se. In order to reduce any bias introduced because only a single RQ measurement was made per time point, we added baseline RQ into the multivariate analyses of RQ changes and starting point as suggested elsewhere [21]. Additionally, to ensure uniform conditions, indirect calorimetry was performed according to strict previously tested protocols [34]. We do appreciate that although RQ might be a potential additional tool to assess differential responses to diet, changes in RQ are of limited clinical relevance as the procedures cannot be performed in clinical settings.

Strengths: This one-phase study with a high retention rate is one of the largest studies to have measured RQ under controlled conditions, for the relatively long period of 6 months. Most studies evaluating the effect of diet on RQ have varied from 24 h to 4 weeks of intervention [22,23,40,44,58], with only a couple lasting 12 weeks [59] or 12 months [24]. Our diet intervention included a monitored/supervised lunch, which is typically the main meal, and the menu was carefully planned by the cafeteria’s kitchen staff, according to the diet group. In addition, extensive nutritional instructions were also given to each group. The use of a 3-Tesla MRI scan to measure body composition and body fat storage constitutes a gold standard tool for the evaluation of fat depots.

This study deepens the understanding of individual metabolic variability and its association with cardiometabolic parameters and body fat. Importantly, at a time when the importance of individual responses is becoming increasingly appreciated, RQ should be taken into account to assess metabolic characteristics and potential different responses to diet. However, the complex interactions between the RQ and diet will require further research.

## 5. Conclusions

Fasting RQ may reflect differences in metabolic characteristics between subjects affecting their potential personal response to diet. Further studies are needed to assess the immediate metabolic response to diet.

## Figures and Tables

**Table 1:** Baseline characteristics of the study participants with full metabolic assessment across diet intervention groups.

		Intervention Groups			
Characteristics	Participants *n* = 159	Low-Fat Diet (*n* = 79)	Mediterranean/Low Carbohydrate (*n* = 80)	Mean Difference (95% CI)	*p*
RQ	0.80 ± 0.04	0.80 ± 0.04	0.81 ± 0.05	0.008 (−0.02–0.006)	0.26
Age (years)	47.7 ± 8.9	48.9 ± 8.9	46.5 ± 8.9	2.4 (−0.3–5.2)	0.09
Male N, (%)	136 (85.5)	66 (83.5)	70 (87.5)		0.47
Weight (Kg)	91.5 ± 14.4	90.6 ± 14.6	92.4 ± 14.2	−1.8 (−6.3–2.7)	0.43
Waist circumference (cm)	106.9 ± 10.3	105.9 ± 9.3	107.9 ± 11.2	−1.9 (−5.1–1.3)	0.23
BMI (Kg/m^2^)	31.1 ± 4.1	30.8 ± 3.9	31.2 ± 4.2	−0.4 (−1.6–0.8)	0.50
RMR (kcal)	1927 ± 318	1898 ± 310	1955 ± 325	−57.5 (−157.1–42.2)	0.25
Body fat (cm^2^)					
Visceral adipose tissue	177.4 ± 69.7	184.6 ± 72.7	170.3 ± 66.4	14.3 (−7.5–36.1)	0.19
Deep Subcutaneous tissue	221.2 ± 76.1	213.7 ± 70.3	228.5 ± 81.1	−14.8 (−38.5–9.0)	0.22
Superficial subcutaneous tissue	148.9 ± 63.9	147.1 ± 65.4	150.7 ± 62.7	−3.6 (−23.6–16.5)	0.72
Hepatic fat (%)	10.85 ± 11.1	10.2 ± 9.9	11.4 ± 12.1	−1.2 (−4.7–2.2)	0.49
Blood cardio-metabolic markers					
Glucose (mg/dL)	107.5 ± 19.6	105.9 ± 14.3	109.2 ± 23.7	−3.3 (−9.4–2.8)	0.29
Insulin (mIU/L)	17.6 ± 11.2	17.6 ± 11.7	17.5 ± 10.7	0.1 (−3.4–3.6)	0.97
HbA1C (%)	5.5 ± 0.5	5.5 ± 0.5	5.6 ± 0.5	0.0 (−0.2–0.1)	0.64
Triglycerides (mg/dL)	73.1 ± 41.1	78.4 ± 45.3	68.1 ± 36.2	10.4 (−2.6–23.3)	0.11
LDL cholesterol (mg/dL)	123.1 ± 30.9	125.8 ± 31.5	120.4 ± 30.2	5.4 (−4.3–15.0)	0.27
HDL cholesterol (mg/dL)	42.9 ± 11.3	42.4 ± 12.2	43.4 ± 10.4	−1.0 (−4.6–2.5)	0.56
Blood pressure (mm Hg)					
Systolic	123.2 ± 15.1	123.4 ± 14.7	123.1 ± 15.5	0.36 (−4.4–5.1)	0.87
Diastolic	79.3 ± 10.5	78.5 ± 10.9	80.1 ± 10.1	−1.5 (−4.8–1.7)	0.35
Reported dietary intake					
Kcal	2845 ± 1075	2841.9 ± 1061.7	2848.8 ± 1095.2	−6.9 (−344.8–331.0)	0.96
Carbohydrates (%)	45.4 ± 7.9	44.3 ± 8.4	46.6 ± 7.3	−1.8 (−4.3–0.6)	0.13
Total fat (%)	34.5 ± 4.8	35.5 ± 5.0	33.5 ± 4.4	1.6 (0.1–3.1)	0.03
Protein (%)	20.1 ± 3.8	20.2 ± 4.0	20.1 ± 3.7	0.06 (−1.1–1.2)	0.91

RQ—respiratory quotient; BMI—body mass index; HbA1C—glycated hemoglobin; HDL—high-density lipoprotein cholesterol; LDL—low-density lipoprotein.

## Appendix A

**Table A1:** Baseline characteristics comparison between participants with and without six-month follow up assessment.

Characteristics	Follow Up *n* = 159	Non-Follow Up *n* = 119	*p*
RQ	0.80 ± 0.04	0.80 ± 0.05	0.299
Age (years)	47.7 ± 8.9	48.1 ± 9.7	0.666
Male *n*, (%)	136 (85.5)	111 (93.2)	0.042
Weight (Kg)	91.5 ± 14.4	91.3 ± 11.9	0.892
Waist circumference (cm)	106.9 ± 10.3	106.3 ± 8.6	0.596
BMI (Kg/m^2^)	31.1 ± 4.1	30.6 ± 3.6	0.325
RMR (kcal)	1927.2 ± 318.5	1902.9 ± 266.3	0.504
Body fat (cm^2^)			
Visceral adipose tissue	177.4 ± 69.7	172.6 ± 61.9	0.552
Deep Subcutaneous tissue	221.2 ± 76.1	209.9 ± 71.7	0.213
Superficial subcutaneous tissue	148.9 ± 63.9	133.9 ± 60.9	0.049
Intramuscular triglycerides (%)	2.4 ± 1.7	2.37 ± 1.4	0.894
Intermuscular fat	9.4 ± 4.5	9.8 ± 4.7	0.513
Renal sinus fat	2.7 ± 1.4	2.6 ± 1.2	0.747
Pancreatic fat	17.1 ± 5.2	17.8 ± 4.9	0.279
Hepatic fat (%)	10.8 ± 11.1	9.2 ± 9.3	0.206
Blood cardio-metabolic markers			
Glucose (mg/dL)	107.5 ± 19.6	107.0 ± 18.9	0.821
Insulin (mIU/L)	17.6 ± 11.2	16.1 ± 8.6	0.227
HbA1c (%)	5.5 ± 0.5	5.5 ± 0.5	0.604
HOMA-IR	4.7 ± 3.6	4.3 ± 2.5	0.195
Triglycerides (mg/dL)	73.1 ± 41.1	71.8 ± 41.3	0.801
LDL cholesterol (mg/dL)	123.1 ± 30.9	121.2 ± 32.1	0.623
HDL cholesterol (mg/dL)	42.9 ± 11.3	43.4 ± 12.1	0.729
Blood pressure (mm Hg)			
Systolic	123.2 ± 15.1	125.6 ± 17.1	0.231
Diastolic	79.3 ± 10.5	80.8 ± 11.0	0.252
Adipokines (ng/mL)			
Leptin	16.7 ± 14.2	11.5 ± 8.9	<0.001
Adiponectin	11.4 ± 10.9	9.5 ± 8.1	0.102

Abbreviations: RQ—respiratory quotient; BMI—body mass index; RMR—Resting metabolic rate; HbA1c—Glycated hemoglobin; HOMA-IR—homeostatic model of insulin resistance; LDL—low-density lipoprotein; HDL—high-density lipoprotein. Continuous variables are expressed as mean ± SD. Differences between groups were calculated using *t*-test. Categorical variables are expressed as Number (%). Differences between groups were calculated using Chi square test. Statistical significance was determined as *p* < 0.05 between groups at baseline.

**Table A2:** Baseline characteristics of the study population across RQ groups.

		RQ Groups		
Characteristics	All *n* = 277	Lower RQ *n* = 137	Higher RQ *n* = 140	*p*
RQ	0.81 ± 0.05	0.77 ± 0.02	0.85 ± 0.04	<0.001
Carbohydrate ratio (%)	36.7%	23.3%	50.2%	
Age (years)	47.9 ± 9.3	48.7 ± 9.2	47.0 ± 9.3	0.135
Male *n*, (%)	246 (88.8)	118 (86.1)	128 (91.4)	0.162
Weight (Kg)	91.4 ± 13.4	91.0 ± 12.6	91.9 ± 14.1	0.572
Waist circumference (cm)	106.7 ± 9.6	106.5 ± 9.9	106.9 ± 9.4	0.727
BMI (Kg/m^2^)	30.9 ± 3.8	31.0 ± 3.8	30.8 ± 3.9	0.609
RMR (kcal)	1916 ± 297	1881 ± 287	1951 ± 303	0.052
Body fat (cm^2^)				
Visceral adipose tissue	175.4 ± 66.4	168.7 ± 58.1	182.1 ± 73.5	0.095
Deep Subcutaneous tissue	216.4 ± 74.3	216.4 ± 77.1	217.1 ± 71.5	0.935
Superficial subcutaneous tissue	142.5 ± 63.0	150.0 ± 74.9	135.7 ± 47.8	0.059
Intra-muscular triglycerides (%)	2.39 ± 1.56	2.58 ± 1.6	2.22 ± 1.5	0.051
Intermuscular fat	9.63 ± 4.6	9.59 ± 4.4	9.69 ± 4.8	0.852
Renal sinus fat	2.68 ± 1.4	2.63 ± 1.4	2.74 ± 1.4	0.493
Pancreatic fat (%)	17.4 ± 5.09	17.31 ± 4.8	17.44 ± 5.3	0.829
Hepatic fat (%)	10.17 ± 10.4	9.98 ± 10.4	10.3 ± 10.4	0.765
Blood markers				
Glucose (mg/dL)	107.3 ± 19.3	107.5 ± 20.5	107.1 ± 18.2	0.863
Insulin (mIU/L)	16.9 ± 10.2	16.59 ± 10.4	17.3 ± 10.0	0.558
HbA1c (%)	5.5 ± 0.5	5.6 ± 0.5	5.5 ± 0.5	0.209
HOMA-IR	4.6 ± 3.2	4.5 ± 3.4	4.6 ± 2.9	0.687
Triglycerides (mg/dL)	72.6 ± 41.1	73.8 ± 45.8	71.4 ± 36.1	0.620
LDL cholesterol (mg/dL)	122.3 ± 31.4	119.2 ± 33.1	125.5 ± 29.4	0.094
HDL cholesterol (mg/dL)	43.1 ± 11.7	43.7 ± 12.2	42.7 ± 11.1	0.489
Blood pressure (mm Hg)				
Systolic	124 ± 16	125 ± 16	123 ± 16	0.207
Diastolic	80 ± 11	80 ± 11	79 ± 10	0.395
Adipokines (ng/mL)				
Leptin	14.5 ± 12.5	15.7 ± 15.1	13.4 ± 9.2	0.134
Adiponectin	10.6 ± 9.9	11.8 ± 10.7	9.5 ± 8.9	0.060

Abbreviations: RQ—respiratory quotient; BMI—body mass index; RMR—Resting metabolic rate; HbA1c—Glycated hemoglobin; HOMA-IR—homeostatic model of insulin resistance; LDL—low-density lipoprotein; HDL—high-density lipoprotein. Groups are divided by median baseline RQ. Continuous variables are expressed as mean ±SD. Differences between groups were calculated using *t*-Test. Categorical variables are expressed as Number (%). Differences between groups were calculated using Chi square test. Statistical significance was determined as *p* < 0.05 between groups at baseline.
